# A Theoretical and Practical Treatise upon Simple and Cancerous Organic Alterations of the Uterus, &c.

**Published:** 1836-10

**Authors:** 


Art. IX.—*Traité Théorique et Pratique sur les Altérations organiques simple et cancéreuses de la Matrice.* Par F. Duparcque, d.m. &c. &c. —*Paris*, 1835. 8vo. pp. 486.

*A Theoretical and Practical Treatise upon simple and cancerous organic Alterations of the Uterus*, &c.

About three years ago the Royal Medical Society of Bordeaux proposed the following question for their prize essay; and to M. Duparcque's treatise was awarded not only the prize, but the very warm approbation of that learned body, for the practical information he imparted upon a subject of no ordinary difficulty.—"To establish the diagnostic characters of different indurations and ulcerations of the neck and body of the uterus: to point out the best mode of treatment for each of them, and to state the cases which require extirpation of the diseased parts."

In this country, prize essays are generally, if not always, the productions of intelligent students, who have read much, and seen but little; but, on the Continent, men of considerable experience do not consider it beneath them to compete for prizes offered by medical societies. M. Duparcque, for example, has evidently had great experience in diseases of the uterus, and he draws principally upon his own resources for the practical illustrations with which his work abounds.

The treatise is divided into two parts. In the first, a rapid sketch is given of the exciting and predisposing causes of chronic diseases of the uterus in general. The origin, and progress of, and the influence of medical treatment upon these diseases, is next considered. The second part contains a separate account of each chronic disease of the uterus, and of cancerous affections; and, lastly, a critical survey is taken of the surgical treatment of cancer of the uterus. M. Duparcque first states briefly and correctly the well known fact, that until the period of puberty the uterus appears to be, as it were, neglected by nature. But, at that important epoch of female life, it almost suddenly becomes exposed to various derangements of function, and various organic diseases, from which it was previously protected by its comparatively inert and passive condition. Five cases are related of chronic diseases of the uterus occurring in virgins, and commencing with suppressed or painful menstruation. Local and general bleeding, warm baths, &c., slowly restored these patients to health. The fourth case is interesting, inasmuch as it shews, that in young females who have never been impregnated, the uterus may, from a simple state of congestion, become so much enlarged as to lead to the suspicion of pregnancy, and that collections of fæces in the large intestines may also create much perplexity to a young practitioner. A girl, æt. fifteen, had menstruated for several months, but before each menstrual period she suffered considerable pain in the hypogastrium, which disappeared when the flow of menses took place. In consequence of her being greatly alarmed, the menses were suppressed, and from this time she suffered from great pains in the belly, frequent vomiting, loss of appetite, &c. Her state became serious, and our author was consulted. Upon examining the abdomen externally, he felt behind the pelvis, and in the hypogastric region, a tumour inclined towards the right iliac fossa, which resembled by its position, form, and volume, the uterus at about the fourteenth week of pregnancy. The tumour was hard, and slight pressure upon it caused pain. In the left iliac region was another tumour, of an irregular oblong shape, and partly within the pelvis. Pregnancy was suspected. No satisfactory examination, per vaginam, could be made, in consequence of these tumours. Upon attempting to examine by the rectum, the finger could not pass further than the sphincter, as the gut was filled with hardened fæces. The patient had now the ordinary symptoms of inflammatory fever: sixteen ounces of blood were taken from the arm; and the rectum was emptied with the handle of a spoon; after which free evacuations were procured by castor oil, and warm injections. The next day there was no tension of the abdomen; the tumour in the left iliac region no longer existed; but the hypogastric tumour remained of the same size, and now appeared more exactly placed on the median line, and within the pelvis. Upon examining, at the same moment, with the index-finger of the right hand in the rectum, which was now empty, and the left hand upon the hypogastrium, M. Duparcque was convinced that the tumour consisted of an enlarged uterus.* The size of the uterus, taken in connexion with the time that had elapsed since the patient had menstruated, confirmed the suspicions of her being pregnant; but, in the case of this young female, there were many reasons for believing she was not pregnant. As she had menstruated, no retention of the menstrual fluid within the uterus could be imagined, and M. Duparcque concluded that the increased size of the uterus was caused by a gorged state of the tissue of the organ; and that she had at first laboured under chronic inflammation of the uterus, which had now become more active. The severe pains she felt, on the slightest pressure, strengthened this opinion. VS. to ℥xij. Next day, twenty leeches to the hypogastrium, which was covered with emollient poultices. Hip bath. Four days afterwards, the tumour in the hypogastrium was much less, and the general symptoms of inflammation had subsided. Pains diminished. Twelve more leeches were applied, and the other remedies continued. In a few days more, the tumour was scarcely to be felt; in about a month, the patient menstruated freely, and her health was completely re-established.

In many points of view this case is interesting, and especially so from the increased size of the uterus in a very young female, from mere congestion, notwithstanding the natural firmness of its tissue.

We find a remark at the end of the detail of the sixth case, which is practically important. For some time after labour, the uterus remains more or less enlarged, and its tissue is engorged with blood; as it is then increased in weight, it may sink towards the vulva, or there may be prolapsus uteri. The relaxation of the vagina as well as of the ligaments of the womb, the consequences of pregnancy and labour, favour this occurrence. If, under these circumstances, the practitioner regards only the displacement of the uterus, without bearing in mind the cause which produces it, and applies a pessary, he does mischief, as the foreign

* The patient was, no doubt, placed on her back, the customary position on the Continent during such examinations, as well as during labour, and certainly, in many cases, the most convenient. If, for the purpose of making such an examination, the patients were placed on the left side, the left index-finger would be introduced into the rectum, while, with the right hand, the uterus would be examined and supported externally.—Rev.

body increases the irritation and flow of blood to the uterus; and thus the foundation may be laid for more serious disease; when small local bleedings, and rest in a horizontal posture, would quickly relieve the patient. M. Duparcque tells us he has known this mistake very frequently committed, by the most celebrated physicians. This we should hardly have thought possible. It is the blunder of carelessness or inexperience.

Upon one subject we perfectly agree with our author. We are quite sure that at least half the fears that books would teach us to apprehend at the "critical age" of women, are more imaginary than real. That at that period certain diseases, which had hitherto remained in a latent state, may assume a more active course, we do not deny; but that any maladies owe their origin to any change that then takes place in the female constitution, we very much doubt. In confirmation of this opinion we may observe, that from very extensive statistical researches made by Moret, Finlaison, and Chateauneuf, it is proved that as many men die, as women, between forty and fifty years of age.

"In forty women," says the author, "of forty or fifty years of age, who were affected with cancerous diseases of the uterus, there were only five in whom the disease had recently appeared. In thirty-three there had been long standing derangement of the menstrual function; dysmenorrhœa, with symptoms of uterine disease, either depending upon labour, miscarriage, or accident. In two cases, which are shortly related, the disease appeared to have been gradually forming from the age of puberty."—"It may be laid down, then, as a general rule, that the 'critical age' is only dangerous to those women who arrive at that period of life with some already existing, but perhaps latent, disease of the uterus."

In this doctrine we fully concur.

The tenth and eleventh cases are examples of great organic disease of the uterus, without any derangement of the general health which could have led to the suspicion of the existence of such a malady. Such instances are by no means rare, and we believe it is pretty generally known that both the ovaria and uterus may undergo almost any extent of organic destruction or alteration, and yet no symptoms exist indicative of such disease. Let us observe, however, that in many of these cases a careful examination of the patient will lead us to detect that which the indefinite nature of her symptoms and complaints might conceal from us.

A young French lady was placed under our care; she complained of nothing but pain in the back, and sickness at the stomach. Her spirits were good, and no apprehensions had existed either in the mind of her medical attendant or friends. "Rheumatism, with a disposition to tic douloureux of the back," was the title her disease had received. But her countenance spoke more correctly than her tongue, and presented every appearance of organic, and especially of uterine disease. Still she complained of nothing but the "rheumatism" in her back. Upon laying the hand upon the abdomen, two large tumours were felt in the region of the ovaria, which were somewhat tender on pressure, but of the presence of which she had not before been conscious. There was also a small indolent tumour in the breast, and others of different size were scattered over the abdomen. The case was clearly one of malignant disease, and the friends were warned that in all probability it would terminate fatally. In about three weeks the patient died; the ovarial tumours having taken an inflammatory action. Upon dissection, both the ovaria were found converted into masses of fungus hæmatodes, and incipient traces of the disease were also apparent on the peritoneal coat of the intestines. Connected with this case, were some other points of much practical importance, but we have entered fully enough into it for our present purpose,—namely, to warn the young practitioner not carelessly to pass over complaints which women may make of severe and long-continued pains in the back and groins, with or without sickness at the stomach. It is true that such symptoms may very frequently depend upon some trifling and passing cause, but they are, not unfrequently, the only signs which attend severe organic disease of the uterus or its appendages, and therefore should never be neglected, if we would avoid the risk of giving erroneous opinions.

The second chapter of the work describes the formation, development, and termination of diseases of the uterus in general. We extract one remark, for the sake of its novelty. The accuracy of the doctrine it inculcates appears to be very questionable.

"It is proved, by an exact account of the operations for cancer performed in the Parisian hospitals during a certain number of years, that relapses are less to be feared in proportion as the disease is of long standing, from whence it may be concluded, contrary to the general opinion, that, so far from hastening the performance of the operation, it is more judicious to wait, until the disease has, as it were, exhausted its power upon the affected organ. It may be added, that by gaining time, we arrive at that age when the vital or organic modifications which constitute the essential predisposing conditions to cancerous diseases are changed or disappear, and that then the disease, confined in future to the part originally attacked, will be no longer disposed to recur, or to form a cancerous diathesis."

Facts like these must come before us in a more satisfactory manner, before we enquire into the solidity of the hypothesis which is brought forward to explain them. At present, without, we hope, being pertinaciously attached to a creed because it has age to recommend it, we must advise the speedy extirpation of cancerous disease, whenever it is practicable, and should not hold the surgeon justifiable who would wait for the aforesaid "change or disappearance of the vital or organic manifestations" to fix the residence of so formidable a foe as cancer.

In the chapter pointing out the best mode of examining patients, for the purpose of detecting uterine disease, the student will find many very useful hints. In this country, examinations by the rectum, by which we can so well ascertain, through the thin coats of the intestine, the form of the cervix uteri, the extent of disease in it, and the condition of the posterior surface of the organ, is too much neglected. In France the speculum is used with the greatest freedom and frequency, and no doubt it often gives the practitioner more decisive information than can be obtained by the most dexterous internal or external manual examination. In the use of this instrument but little pain is given in most cases, but the mental pain which most women must suffer from its employment will probably prevent it from ever being commonly used in this country. For the performance of certain operations on the uterus the use of the speculum is absolutely necessary.

In the second part of the work M. Duparcque treats, in different chapters, of each organic disease to which the uterus is liable, and of every form of uterine disease cases are given to illustrate their progress, and the effects of remedies. We shall not enter into the detail of these cases, but confine ourselves to the chief practical remarks. The author observes that it is by no means uncommon to find the cervix uteri studded with fleshy growths of different form and consistence; their development is generally slow, and their presence causes no particular disturbance. Portal makes the same remark. He found these growths on the cervix uteri in many instances, and their existence had not been suspected during life. These tumours are generally insensible, soft, and bleeding, and give rise to a moderate sero-mucous discharge. Sometimes they increase to a very large size, become firm, and are attended with a profuse serous, mucous, or bloody discharge. And in this state they are not unfrequently, according to Breschet, mistaken for cancer.

If there is any one practice more frequently abused, and more empirically employed, than another, it is that of the indiscriminate use of astringent medicines, or injections in cases of uterine hemorrhage. Upon this subject M. Duparcque gives a useful lesson. The use of astringents in cases of uterine hemorrhage demands especial care. It is true they may suppress the discharge, but they do not always diminish the cause which gives rise to it. During the employment of astringents a congested state of the uterus may not only continue, but be positively aggravated. And most physicians must have seen cases in which acute inflammation of the uterus was provoked by the use of astringent remedies. Their use then should not be ventured upon until, 1st, any local determination of blood to the uterus has been relieved by proper treatment; 2d, or until the hemorrhage appears to be the result of atony, or relaxation of the tissue of the uterus, and there is no proof that the capillary circulation is too active. When uterine hemorrhage has continued for some time, and general debility is produced, then cold and astringents are powerful remedies. M. Duparcque thinks well of the ergot of rye in cases of hemorrhage which arise from a congested state of the uterus. We have always been of opinion that the attempts which have been made to establish a diagnosis between different diseases of the uterus, by attending to the nature and appearance of the "discharges" were at least very fanciful, and we find the author agrees with us. "The discharges from the vagina can form no diagnostic mark of the existence or nature of induration of the uterus." Of one remedy, which is much extolled, we ourselves know nothing; but our list of diuretic remedies is so scanty that we must not omit to add to it upon the respectable authority of M. Duparcque: In a case of ovarial enlargement, with considerable effusion, he was on the point of puncturing the abdomen, when he determined to try the *Pareira brava*, the powerful diuretic properties of which he had before ascertained. He gave it in the form of decoction, two ounces to a pint of water, reduced to a third by boiling. "So active were the effects of the remedy that no other treatment was required. After a few cups of the decoction had been given, the urine flowed in extraordinary abundance: a large chamber-pot being filled twice or three times in the twenty-four hours. The patient soon recovered.*

* The Pareira brava, like many other remedies, has been praised, neglected, and praised again. Helvetius regarded it as an excellent lithontriptic, and was certain that

M. Duparcque directs our particular attention to the practice of applying leeches to the cervix uteri. He affirms that he has derived the happiest effects from it in cases of induration of the fundus or cervix uteri, even when every symptom rendered it probable that the disease was of a schirrous nature, and had before resisted all ordinary treatment. "The rapidity with which resolution took place afterwards has been such as should have been witnessed in order to guard us against the imputation of excessive partiality for the practice, or exaggeration of the facts. The first effect of the application of leeches to the cervix uteri, which constantly takes place, even if the disease is of a cancerous and incurable nature, is to calm, as if by enchantment, the violent pains in the back, and those painful symptoms which are the ordinary accompaniments of organic diseases of the uterus. The number of leeches must be proportioned to the degree of induration, the severity of the local inflammation, and the strength of the patient. This mode of abstracting blood produces much less debility than general bleeding, and may therefore be employed when the latter would be prejudicial, as for example, in the last stage of cancer. The most dreadful symptoms of cancer of the uterus result less from the disease itself, than from inflammation, which it causes. Thus schirrous tumours, like softened encephaloid masses, do not ulcerate until inflammation has taken place. And it is at this period that those dreadful pains arise which characterize the progress of cancer, and perhaps for the first time denote their existence. In these cases, leeches applied to the tumour arrest the process of inflammation, suspend the violent pains it had caused, and thus the fatal progress of the disease is arrested. In cancerous ulcerations, observation proves, that the disease does not invade the neighbouring parts until after inflammation has taken place in them; and in these cases leeches applied to the part relieve the sufferings of the patient, and may suspend the destructive progress of the ulceration. Thus, then, leeches applied to the diseased part promote a cure, or furnish in incurable cases one of the most powerful palliations that art possesses. They are not only applicable when the cervix alone is indurated, but are equally advantageous when the whole of the uterus is affected."

When the uterus is low enough down for the cervix to be perceived by separating the labia, leeches may be easily applied. When the uterus is in its natural situation, the speculum must be used, to enable us to apply the leeches to the cervix. When the instrument is applied so that the circumference of its opening exactly and exclusively embraces the cervix, the vagina must be freely washed out with water injections, to cleanse the parts from discharges which would prevent the leeches from biting. The leeches are to be introduced into the speculum, and pushed up by a roll of lint to prevent their returning: the lint being removed when they have fixed. The bites of the leeches produce little pain. M. Duparcque has observed that when the induration appeared to be of a schirrous nature, the punctures made by the leeches bled but little, and that, on the contrary, when the cervix was more inflamed than indurated, the bleeding was more profuse, and sometimes even sufficiently so to it would render unnecessary the operation for lithotomy. Its diuretic properties have been extolled by many. Mérat says (Dict. des Sc. Méd. 39-288) that it may be safely erased from our medical catalogue. *Rev.*

create anxiety. Twelve is the greatest number of leeches ever applied in this manner by the author, and six or eight are generally sufficient.

"The unlooked for success," he says, "that I have obtained from the application of leeches to the cervix uteri in cases of simple induration, and in more serious diseases of the organ, justify me in regarding the practice as superior to all those which have been advised in these fearful cases. The cure of some cases, and the relief of others, have been greatly owing to this practice." Other means, however, are not to be neglected; and in most cases one or more general bleedings are necessary before leeches are thus applied.

We have thought it right to put our readers in possession of M. Duparcque's statements regarding a mode of practice which, as far as we know, has not been tried in this country. In cases of inflammation, or induration of the uterus, too, M. D. speaks highly of tartar emetic frictions upon the inside of the legs, arms, and chest; and several cases are related, from which it would appear that much benefit was derived from this plan. The strength of the ointment recommended is ʒj. of Ant. Tart. to ℥j. of lard. About ʒss. of this ointment is used for each friction.

The seventy-seventh case is related principally for the purpose of proving the benefit that may be derived in alleviating the pain of an incurable cancerous disease, from the endermic method of applying acetate of morphium to a blistered surface, after opium had been administered in large doses internally without benefit. We have seen enough of this mode of practice to justify a regret that it is not more frequently tried by English physicians.

Upon the subject of amputation of the cervix uteri, we find some very sensible remarks, which, together with those of Boivin and Dugés,* will, we trust, curb the confidence, to use no harsher term, of those Continental surgeons who have so frequently performed this operation. M. Duparcque assures us, of that indeed which we are quite prepared to believe from an attentive perusal of many of these recorded operations, that, judging from facts, he is persuaded that amputation of the cervix uteri has been very often performed when it was at least useless, and that among the many anatomical preparations which have been triumphantly exhibited, he and others have seen several in which there was no appearance of disease that could justify so formidable a proceeding.

Of M. Duparcque's work we may, in all sincerity, say that it is very creditable to himself, and cannot fail to be useful to students and practitioners. The size of the volume is certainly rather unnecessarily enlarged by the detail of eighty-seven cases, of which most might fairly have been condensed, and many safely omitted. The more recent work of Mdme. Boivin and Dugés upon the same subject, which has been so well condensed, translated, and enriched with copious notes, by Dr. Heming, for the convenience of the English reader, is certainly superior as a general practical treatise; but, admitting this, M. Duparcque's work contains too much valuable matter, and too many original views, to be passed over without loss by any who are prosecuting the very important subjects upon which he treats.

* Traité pratique sur les Maladies de la Matrice, &c.

